# Human Nek6 is a monomeric mostly globular kinase with an unfolded short N-terminal domain

**DOI:** 10.1186/1472-6807-11-12

**Published:** 2011-02-14

**Authors:** Gabriela V Meirelles, Júlio C Silva, Yuri de A Mendonça, Carlos HI Ramos, Iris L Torriani, Jörg Kobarg

**Affiliations:** 1 Laboratório Nacional de Biociências, Centro Nacional de Pesquisa em Energia e Materiais, Campinas, SP, Brazil; 2 Departamento de Bioquímica-Programa de Pós-graduação em Biologia Funcional e Molecular, Instituto de Biologia, Universidade Estadual de Campinas, 13083-970 Campinas, SP, Brazil; 3 Instituto de Química, Universidade Estadual de Campinas, Campinas, SP, Brazil; 4 Laboratório Nacional de Luz Síncrotron, Centro Nacional de Pesquisa em Energia e Materiais, Campinas, SP, Brazil; 5 Instituto de Física "Gleb Wataghin", Universidade Estadual de Campinas, Campinas, SP, Brazil

## Abstract

**Background:**

The NIMA-related kinases (Neks) are widespread among eukaryotes. In mammalians they represent an evolutionarily conserved family of 11 serine/threonine kinases, with 40-45% amino acid sequence identity to the *Aspergillus nidulans *mitotic regulator NIMA within their catalytic domains. Neks have cell cycle-related functions and were recently described as related to pathologies, particularly cancer, consisting in potential chemotherapeutic targets. Human Nek6, -7 and -9 are involved in the control of mitotic spindle formation, acting together in a mitotic kinase cascade, but their mechanism of regulation remain elusive.

**Results:**

In this study we performed a biophysical and structural characterization of human Nek6 with the aim of obtaining its low resolution and homology models. SAXS experiments showed that hNek6 is a monomer of a mostly globular, though slightly elongated shape. Comparative molecular modeling together with disorder prediction analysis also revealed a flexible disordered N-terminal domain for hNek6, which we found to be important to mediate interactions with diverse partners. SEC-MALS experiments showed that hNek6 conformation is dependent on its activation/phosphorylation status, a higher phosphorylation degree corresponding to a bigger Stokes radius. Circular dichroism spectroscopy confirmed our *in silico *predictions of secondary structure content and thermal stability shift assays revealed a slightly higher stability of wild-type hNek6 compared to the activation loop mutant hNek6(S206A).

**Conclusions:**

Our data present the first low resolution 3D structure of hNek6 protein in solution. SAXS, comparative modeling and SEC-MALS analysis revealed that hNek6 is a monomeric kinase of slightly elongated shape and a short unfolded N-terminal domain.

## Background

Mitotic progression and assembly of the bipolar mitotic spindle are regulated by several serine/threonine protein kinases, including members of the cyclin-dependent kinase (Cdk), Polo-like kinase (Plk), Aurora, and NIMA-related kinase (Nek) families [1-4]. The founding member of Nek family, the NIMA kinase of *Aspergillus nidulans*, contributes to multiple aspects of mitotic progression including the timing of mitotic entry, chromatin condensation, spindle organization and cytokinesis. Mammals contain a large family of eleven Neks, the catalytic domain of which is evolutionarily related to that of NIMA [4]. Nek2 has a central role in centrosome maturation and disjunction [5], whereas Nek1 and Nek8 have been proposed to contribute to ciliary function [6,7]. Besides Nek2, Nek1, -6, -7 and -9 were also described to participate in centrosomal regulation [7-11]. Nek6, Nek7 [9] and Nek9 [12] are involved in the control of mitotic spindle formation, acting together in a mitotic kinase cascade, with Nek9 being upstream of Nek6 and Nek7 [13]. Nek kinases are also described as related to pathologies, particularly cancer, presenting thereby interesting potential chemotherapeutic targets [14-20]. Recently, hNek6 was described to have its transcript, protein, and/or kinase activity levels highly elevated in a number of tumors and human cancer cell lines, indicating an important role for hNek6 in tumorigenesis [21-24].

Structurally, Neks in general are characterized by having a conserved N-terminal catalytic domain, followed by a nonconserved C-terminal regulatory domain that varies in size and structure. However, Nek6 and Nek7 are significant exceptions to this, in that they are the smallest of the kinases and consist only of a catalytic domain with a relatively short N-terminal extension [4]. Although they share significant similarity with each other, being ~86% identical within their catalytic domains, the N-terminal extensions are not conserved, and it has been suggested that they may play a role in differential regulation of the kinases [25].

The mechanisms of regulation of hNek6, -7, and -9 kinases are currently unknown, and elucidating this pathway would provide relevant knowledge on early mitotic events as well as new hints for drug design and cancer therapy. However, hNek2 and hNek7 are the only NIMA-related kinases for which structures have been reported [26-28]. In this context, we present here the first low resolution three-dimensional structure of hNek6 protein in solution. SAXS experiments, together with SEC-MALS and comparative molecular modeling revealed a monomeric mostly globular, though slightly elongated conformation for hNek6, with a flexible disordered N-terminal domain.

## Results and Discussion

### Human Nek6 is predicted to be phosphorylated at various sites and has an unfolded short N-terminal domain

**Figure 1:**
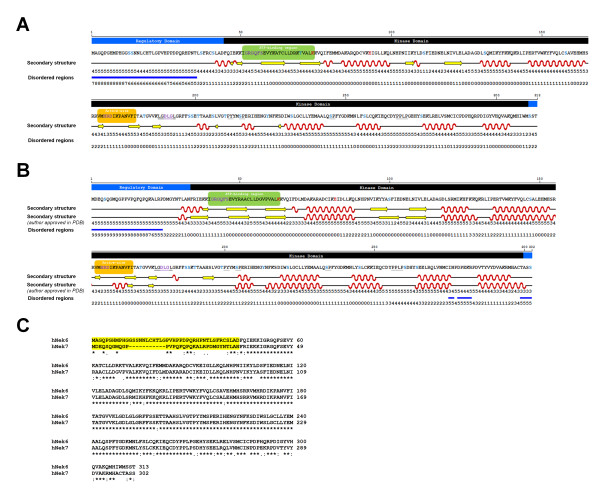
**Human Nek6 and Nek7 structure predictions and sequence aligment**. Consensus of predicted secondary structure for Nek6 (A) and Nek7 (B), is represented by helices (red waves), strands (yellow arrows) and coils (black lines); it was generated from 5 different predictions and the consensus score for each amino acid (1-5) is shown (scores 1-2 represent ambiguous predictions). The consensus of predicted disordered regions was generated from 9 predictions; it is represented by blue bars when the consensus score (0-9) is from 5 to 9. The N-terminal regulatory and C-terminal kinase domains are depicted and two key signatures within the kinase domain are in colored boxes as follows: ATP-binding region, green; Ser/Thr kinases active-site, orange. The conserved glycine-rich sequence, HRD and DLG motifs are in purple, and the conserved residues K^74 ^(β3 strand) and E^93 ^(αC helix) are in red. The putative nuclear export signal LGDLGL based on la Cour et al., 2004 [63], is underlined and the putative WW domain binding motifs PY and pSP based on Ingham et al., 2005 [64], as well as the PPLP motif experimentally described for hNek6 by Lee et al., 2007 [65], are indicated by dotted lines. Putative phosphorylation sites were predicted by NetPhosK 1.0 software and are indicated in blue; S^206 ^in hNek6 is also indicated because of previously described phosphorylation of this residue by hNek9 [13]. These motifs are also present in Nek7 amino acid sequence. The author-approved secondary structure of hNek7 in PDB (2WQM) is also depicted. (C) Primary sequence alignment between human Nek6 (NP_055212.2) and Nek7 (NP_598001.1) using Clustal W2. The N-terminal regulatory domain is highlighted in yellow.

Human Nek6 amino acid sequence was analyzed considering its secondary structure, disordered regions, conserved motifs and putative phosphorylation sites by upstream kinases, resulting in a linear representation of its main structure predictions (Figure 1A). These analyses were also performed for hNek7, for which the crystallographic structure was recently determined [28], in order to validate our results (Figure 1B).

Our consensus of secondary structure was scored by the number of times (one to five times) the predicted secondary structure element (α-helices, β-strands or coils) scored positive from five predictions using different databases: **PredictProtein/Prof **[29], **PSIPRED **[30], **SSpro **[31], **SOPMA **[32] and **GOR4 **[33]. In summary, the secondary structure analysis suggested that hNek6 was composed of approximately 34% α-helices, 12% β-strands and 54% coils. Our hNek7 consensus of predicted secondary structure is 80% identical to the author-approved secondary structure in PDB (2WQM) (Figure 1B).

In the case of the disordered regions predictions, our consensus was obtained following the same criteria as for the secondary structure predictions, except that we used here nine different databases: **FoldIndex **[34], **GlobPlot Russell/Linding **[35], **PONDR VL-XT **[36], **DISpro **[37], **IUPred **[38], **DisEMBL Hot-loops**, **DisEMBL Remark-465**, **DisEMBL Loops/coils **[39], and **VSL2B **[40]. From this analysis, we were able to identify a short high scored segment of disorder covering the majority of hNek6 N-terminal extension before its catalytic domain, which we are calling here the regulatory domain. This characteristic is also present in our hNek7 consensus of disorder predictions and in its crystal structure [28], where amino acids 1-19 are missing residues (due to a flexible region) and amino acids 20-23 are coils. Notably, we found that hNek6 unfolded short N-terminal region is important to mediate interactions with diverse partners [41] and, since hNek6 and hNek7 are similar in their catalytic domain sequences (~86% identity), but different in their N-terminal extensions (~20% identity) (Figure 1C), it is possible that both proteins depend on their disordered N-terminal domain to regulate the interactions with specific/different partners.

For phosphorylation analysis, NetPhosK [42] and NetPhos [43] databases were used to predict phosphorylation sites (Table 1), and together with *in vitro *and *in vivo *phosphorylation data [13], they were used to assign tyrosine, threonine and serine residues as putative phosphorylation sites for hNek6 and hNek7 (Figure 1). This analysis shows a variety of high score phosphorylation predictions for hNek6. Interestingly, there are four predicted sites localized in hNek6 N-terminal domain (serine residues 13, 14, 37 and 41), and one of them (Ser^37^) also described to be phosphorylated *in vivo *[13], suggesting that this is an important phosphorylation-regulated region.

**Table 1:** Prediction of putative phosphorylation sites in human Nek6.

**Residue**^**a**^	**Putative Upstream Kinase**^**b**^	Predictor (Score)
S^13^	PKA	NetPhosK (0.63)
S^14^	PKA	NetPhosK (0.52)
S^14^	Cdc2	NetPhosK (0.53)
S^37^	PKC	NetPhosK (0.82)
S^41^	PKC	NetPhosK (0.56)/NetPhos (0.72)
S^41^	PKA	NetPhosK (0.65)/NetPhos (0.72)
T^70^	PKC	NetPhosK (0.79)
S^111^	CKII	NetPhosK (0.64)
S^131^	DNAPK	NetPhosK (0.62)
S^131^	ATM	NetPhosK (0.68)
S^158^	CKII	NetPhosK (0.53)
S^158^	Cdc2	NetPhosK (0.51)
T^183^	PKC	NetPhosK (0.65)
S^198^	PKA	NetPhosK (0.56)/NetPhos (0.79)
S^198^	Cdc2	NetPhosK (0.50)/NetPhos (0.79)
S^199^	CKII	NetPhosK (0.50)
S^199^	Cdc2	NetPhosK (0.55)
T^201^	PKC	NetPhosK (0.73)
S^206^	CKI	NetPhosK (0.53)/NetPhos (0.99)
T^210^	GSK3	NetPhosK (0.51)
T^210^	CDK5	NetPhosK (0.55)
S^215^	GSK3	NetPhosK (0.50)/NetPhos (0.99)
S^215^	Cdk5	NetPhosK (0.59)/NetPhos (0.99)
Y^224^	EGFR	NetPhosK (0.55)/NetPhos (0.72)
S^232^	PKA	NetPhosK (0.67)
S^245^	p38MAPK	NetPhosK (0.50)
S^245^	Cdk5	NetPhosK (0.55)
S^256^	CKI	NetPhosK (0.54)
S^256^	PKC	NetPhosK (0.50)
S^275^	CKII	NetPhosK (0.52)
S^311^	PKC	NetPhosK (0.66)

^a ^Putative phosphorylated residues predicted with the highest scores by NetPhosK, which may additionally be predicted by NetPhos server.

^b ^Kinases as predicted by NetPhosK to phosphorylate the corresponded residue in hNek6 sequence [Protein Kinase A, C or G (PKA, PKC and PKG); Cell division cycle 2 (Cdc2); Casein Kinase I or II (CKI and CKII); DNA-dependent Protein Kinase (DNAPK); Ataxia Telangiectasia Mutated (ATM); Ciclin-dependent kinase 5 (Cdk5); Glycogen synthase kinase 3 (GSK3); Epidermal Growth Factor Receptor (EGFR); Mitogen-Activated Protein Kinase p38 (p38MAPK)].

### Secondary structure analysis

**Figure 2:**
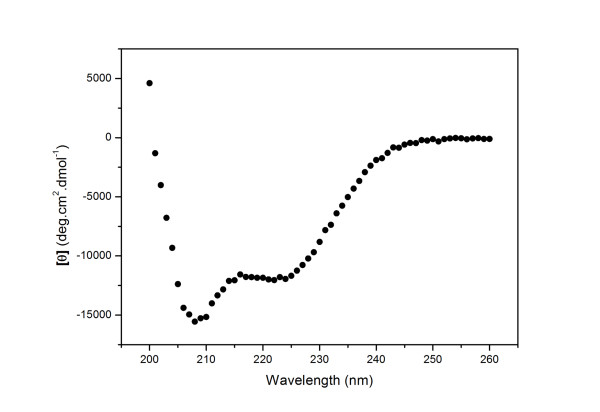
**Far-UV Circular Dichroism spectra of recombinant hNek6wt fused to a 6xHis tag**. Residual molar ellipticity was measured from 200 to 260 nm at a protein concentration of 5.8 μM, in 50 mM Phosphate buffer, pH 7,5 with 300 mM NaCl at 4°C using a Jasco J-810 spectropolarimeter.

The secondary structure content of hNek6 was analyzed by Circular Dichroism (CD) spectroscopy. Figure 2 shows the CD spectra of recombinant wild-type hNek6 fused to a 6xHis-tag recorded at 4°C. Purified protein presents negative ellipticity in the far-UV, with minima at 208 (-15567 deg cm^2 ^dmol^-1^) and 222 nm (-12053 deg cm^2 ^dmol^-1^). This spectrum is typical of many globular proteins [44] and suggests a high content of α-helices, since this secondary structure is characterized by minima around 208 and 222 nm. Deconvolution of the CD spectrum using the CDNN software [45] indicated approximately 41.7% of α-helices, 13.2% of β-strands, 15.7% of beta-turns and 25.8% of random coils. Deconvolution using another software, K2d [46], indicated a similar amount of secondary structure elements: approximately 41% of α-helices, 17% of β-strands and 42% of coils. We also estimated the quantity of α-helix structure by the evaluation of the CD spectrum signal at 222 nm, according to Corrêa and Ramos, 2009 [47], resulting in 38.6% of α-helices. Compared to our predictions, wild-type hNek6 showed a very similar content of α-helices (~34%), β-strands (12%) and coils (54%). In conclusion, both the *in silico *prediction and the experimentally derived data are in reasonable agreement, since they demonstrate an expected high α-helical content for hNek6.

### Comparative molecular modeling of human Nek6

The absence of a three-dimensional structure of hNek6 and the increasing interest in studying Nek proteins within the context of drug design strategies prompted us to construct a homology model for the aforementioned protein. Here, we are interested in the activation loop mutant hNek6(S206A). The activation loop is a centrally located loop, typically 20-30 residues in length, that provides a platform for the peptide substrate. Activation of most protein kinases usually requires phosphorylation of a residue in this loop, which leads to a rearrangement of the loop and increase in enzymatic activity [48]. In hNek6, S206 is an important residue, which phosphorylation leads to an increase in the activation status of the kinase [13,41]. The activation loop has the capacity to undergo large conformational changes when the kinase switches between inactive and active states, adopting distinct conformations in different kinases when they are inactive (unphosphorylated activation loop), a fact that has recently been exploited to great medical benefit [49] and which makes our hNek6 mutant an interesting target to be studied.

**Figure 3:**
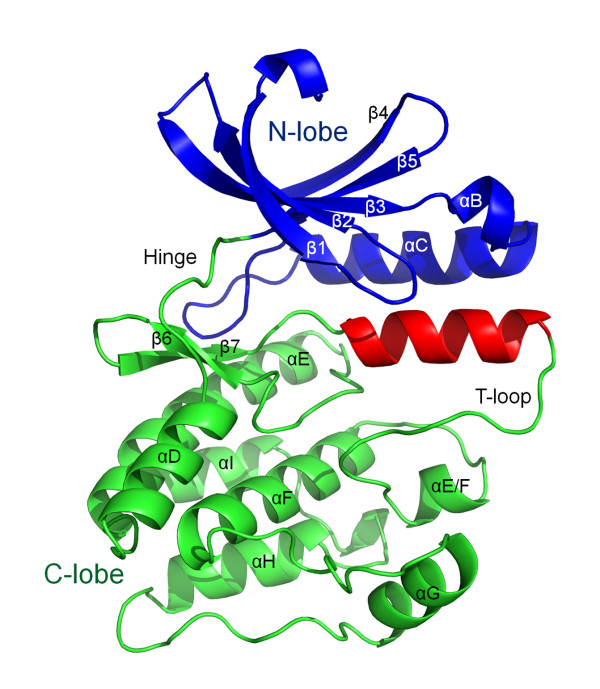
**Comparative molecular modeling of hNek6(S206A) based on hNek7 crystal structure**. Resulting homology model obtained using the structure deposited in the protein data bank under the code 2WQM. The N and C lobes are colored blue and green, respectively, and the predicted α-helix following the conserved DLG motif is shown in red. The hinge region and the activation loop (T-loop) are depicted.

To obtain a homology model of the hNek6 protein, the crystallographic structure of hNek7, available in the Protein Data Bank (PDB: 2WQM) [28], was used as a template. Both proteins share about 77% identity in primary sequence alignment, being ~86% identical in their catalytic domain sequences (Figure 1C). Several homology/comparative modeling tools were used as described in the "Methods" section of this article. In order to choose the best predicted model, stereochemistry quality analyses were done to check for φ and ψ torsion angles using the Ramachandran plots. A comparison of the results indicated that the model generated by SWISS-MODEL [50] is more acceptable than those generated by the other programs (more amino acids in the most favourable regions and less in the disallowed regions). The SWISS-MODEL homology model is shown in Figure 3. The Ramachandran plot (Additional file 1, Figure S1A) showed 86.6% residues in the most favourable regions, 12.1% in additional allowed regions, 0.8% in generously allowed regions and 0.4% (only 1 amino acid) in a disallowed region. As compared to the 2WQM template, these values were 90.8%, 8.8%, 0.4% and 0.0%, respectively. It is important to keep in mind that the template has relatively long regions (one of them consists of 18 amino acids) where the phases could not be solved by X-ray crystallography. Consequently, the homology modeling may not be so accurate in these regions, although the high identity of the target-template sequences makes the whole model plausible. The results revealed that the majority of the amino acids are in a φ-ψ distribution consistent with right handed α-helix and reliable to be a good quality model (Additional file 1, Figure S1A). More details of the validation of the predicted structure results and its quality assessment using PROSA [51,52] are displayed in the Additional Material section (Additional file 1, Figure S1B and S1C). No abnormalities were observed in the validation process, which indicated a good model for the protein.

Our hNek6(S206A) model generated by SWISS-MODEL [50] shows a short region of α-helix composed of twelve residues (G^192^LGRFFSSETTA^203^) following the conserved DLG motif, with high score (Figure 3). A helical structure following the DFG/DLG motif is also present in hNek2(T175A) structures (PDB: 2JAV, 2W5H and 2W5B) [26,27] and in other kinase families, such as inactive forms of both the EGFR kinase [53] and Src/Hck [54]. Therefore, although the activation loop is missing in the electron density map of hNek7, a short helical structure is possibly present in hNek6(S206A), which was predicted in the model generated by SWISS-MODEL [50].

### Human Nek6 is a monomeric mostly globular, though slightly elongated protein in solution, as revealed by SAXS

**Figure 4:**
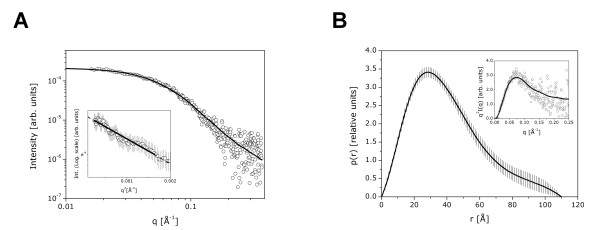
**Small Angle X-Ray Scattering (SAXS) of recombinant hNek6(S206A)**. (A) Experimental SAXS curve from hNek6(S206A) (open circles) and the theoretical fitting (solid line) calculated using the program GNOM. *Inset: *Guinier region and linear fit. (B) Pair distance distribution function *p(r)*. *Inset: *Kratky plot (*q*^*2*^*I(q) vs. q*) of the intensity curve.

To study hNek6 molecular structure, in addition to our homology modeling, we also performed SAXS (Small Angle X-ray Scattering) experiments for the recombinant 6xHis-hNek6(S206A). SAXS is a very useful technique for the determination of overall size, shape and oligomerization status of the macromolecules in solution [55-58]. Figure 4A shows the corrected and normalized experimental scattering curve and theoretical fitting of data by using the program GNOM [59]. The Guinier region providing an *R*_g _value of 32.0 ± 1.0 Å is shown in the inset. The *p*(r) function resulting from these calculations is shown in Figure 4B, with an inset showing the Kratky representation of the intensity curve. The Kratky plot indicates a slightly globular conformation for 6xHis-hNek6(S206A) in solution, although, as expected, the same plot also indicated the presence of flexible regions in the structure, possibly the N-terminal region and the activation loop. The *p(r) *function shows that the protein conformation is slightly elongated. The maximum dimension (*D*_max_) value obtained was approximately 110 Å and the *R*_g _value calculated from the *p*(r) function was 32.4 ± 0.8 Å, in close agreement with that calculated from the Guinier approximation. Using BSA as a standard sample, the molecular mass of 6xHis-hNek6(S206A) estimated from the SAXS data was ~42 kDa. This value indicates that the protein is a monomer in solution, since the theoretically calculated molecular mass of the monomer was ~38 kDa (calculated from the amino acid sequence using ProtParam tool [60]). The molecular mass and consequently the monomeric nature were also confirmed by analytical size-exclusion chromatography coupled to multi-angle light scattering (SEC-MALS).

**Figure 5:**
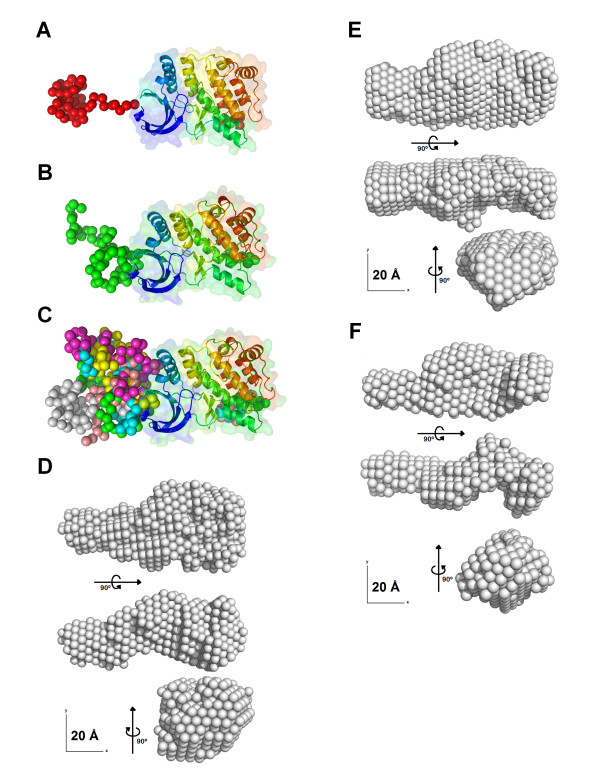
**Low-resolution models of hNek6(S206A) obtained from the SAXS data using a combination of *ab initio *calculations and rigid body modeling**. (A and B) Two typical models selected from the set of 10 resulting models. (C) Superposition of all 10 models obtained in different and independent runs of the program BUNCH. (D) Three orthogonal views of the average molecular envelope of the 10 low resolution BUNCH models. (E) Three orthogonal views of the *ab initio *dummy atoms model. (F) Three orthogonal views of the *ab initio *dummy residues model.

The low-resolution models obtained from the SAXS data by the combination of *ab initio *calculation and rigid body modeling methods are presented in Figure 5. The calculated homology model of the mutant hNek6(S206A) was used in the rigid body calculation. We displayed two typical models of the set of results (Figure 5A and 5B) and a superposition of all 10 models (Figure 5C) obtained in different and independent runs of the program BUNCH [61]. In spite of the flexibility of the N-terminal region, the NSD values of the pairwise comparison of the models obtained ranged from 0.96 to 1.20, which shows the stability of the independent calculations. In order to further compare the resulting models with the information contained in the SAXS curve, we also calculated one average molecular envelope of the 10 models (Figure 5D) and compared with the filtered average models obtained from the two sets of 10 purely *ab initio *model calculations (Figure 5E and 5F).

### Analytical size-exclusion chromatography reveals variations in human Nek6 conformation dependent on its phosphorylation status

**Figure 6:**
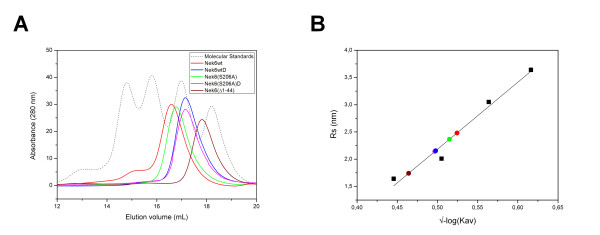
**Analytical size-exclusion chromatography of recombinant hNek6 variants fused to a 6xHis tag**. (A) Chromatographic profile of hNek6 variants (colored lines) and a mixture of standard proteins (dashed line). (B) Linear fit of known Stokes radii (*R*_s_) as a function of the measured partition coefficient (*K*_av_). The colored circles show the partition coefficient of hNek6 variants, and the black squares, the partition coefficients of the standards. The circles colors correspond to the same colors depicted in (A). The proteins were eluted in 50 mM HEPES pH 7.5, 5 mM sodium phosphate, 600 mM NaCl, 5% glycerol, as homogeneous species at different elution volumes (V_e_) using an analytical Superdex 200 10/300 GL column. Conalbumin, ovalbumin, carbonic anhydrase and ribonuclease were used as Stokes radii standards. (wt: wild-type, D: dephosphorylated).

Analytical Size-Exclusion Chromatography (SEC) was performed for five variants of recombinant hNek6 fused to a 6xHis tag: wild-type hNek6 (6xHis-hNek6wt), activation loop mutant hNek6(S206A) (6xHis-hNek6(S206A), kinase domain of wild-type hNek6 (6xHis-hNek6(Δ1-44)), and dephosphorylated wild-type and mutant hNek6 (6xHis-hNek6wtD and 6xHis-hNek6(S206A)D). Interestingly, the dephosphorylated wild-type and mutant proteins, which were co-expressed with lambda phosphatase, were eluted at equal elution volumes (Figure 6A), showing the same Stokes radius of ~2.1 nm, while the more phosphorylated wild-type hNek6 showed a larger radius of ~2.6 nm and the partially phosphorylated mutant hNek6(S206A) showed an intermediate radius of ~2.4 nm (Figure 6B, Table 2). As expected, 6xHis-hNek6(Δ1-44) showed the smallest radius of ~1.8 nm. Wild-type hNek6 elution curve also showed a smaller peak corresponding to a population of higher molecular weight, possibly due to aggregates. It is interesting to compare the Stokes radius estimated by SEC for 6xHis-hNek6(S206A) (~2.4 nm) with the radius of gyration determined by SAXS (~3.2 nm). The resulting ratio *R*_g_/*R*_s _for this protein is ~1.3 and, as *R*_g_/*R*_s _ratios are reported to vary from 0.78 for homogeneous spheres, up to values nearing 2 for extended coils and prolate ellipsoids [62]. This reinforces our results that hNek6 has a slightly elongated conformation, with a flexible unfolded N-terminal domain contributing to this shape.

**Table 2:** Recombinant hNek6 Stokes radii (*R*_s_) determined by analytical size-exclusion chromatography.

Protein	***R***_**s **_**(nm)**	**V**_**e **_**(mL)**^**a**^	**Apparent *R***_**s **_**(nm)**^**a**^
Conalbumin	3.6	14.9 ± 0.1	3.5 ± 0.2
Ovalbumin	3.0	15.8 ± 0.1	2.9 ± 0.0
Carbonic Anhydrase	2.0	16.9 ± 0.1	2.2 ± 0.0
Ribonuclease	1.6	18.2 ± 0.0	1.4 ± 0.1
Nek6wt		16.4 ± 0.3	2.6 ± 0.1
Nek6wtD		17.1 ± 0.0	2.1 ± 0.0
Nek6(S206A)		16.7 ± 0.2	2.4 ± 0.0
Nek6(S206A)D		17.1 ± 0.0	2.1 ± 0.0
Nek6(Δ1-44)		17.6 ± 0.3	1.8 ± 0.1

^a ^S.E. of two measurements at different buffers.

SEC was also coupled to MALS (Multi-Angle Light Scattering), which is an useful technique to measure the weight average molecular masses (*M*_w_) of the eluted proteins, as described in the Methods section. As expected, wild-type and mutant hNek6, dephosphorylated or not, showed a *M*_w _of ~38 kDa, while the kinase domain showed a *M*_w _of ~33 kDa (Table 3, Additional file 1, Figure S2). This corroborates our experimental data from SAXS for 6xHis-hNek6(S206A) and the theoretically calculated molecular masses for all hNek6 variants, showing that hNek6 is a monomer in solution.

**Table 3:** Recombinant hNek6 weight average molecular masses (*M*_w_) determined by SEC-MALS and melting temperatures (*T*_m_) during thermal shift denaturation.

Protein	**Pred. *M *(kDa)**^**a**^	***M***_**w **_**(kDa)**^**b**^	**Apparent *T***_**m **_**(°C)**^**c**^
Nek6wt	37.7	38.4 ± 0.6	39.5 ± 0.1
Nek6wtD	37.7	37.8 ± 2.2	40.8 ± 0.1
Nek6(S206A)	37.7	38.1 ± 2.1	38.0 ± 0.1
Nek6(S206A)D	37.7	38.4 ± 3.3	36.8 ± 0.3
Nek6(Δ1-44)	33.2	33.3 ± 1.6	41.0 ± 0.2

^a ^Molecular masses predicted by ProtParam tool [60].

^b ^S.E. of two measurements at different buffers.

^c ^S.E. of three measurements.

These results suggest that, although having the same molecular mass of ~38 kDa, wild-type hNek6 is purified from bacteria more phosphorylated than its mutant variant, mainly because of their different activation/autophosphorylation status, as described by Meirelles et al. [41], and these different phosphorylation degrees may cause changes in protein conformation and compactness, resulting in changes in their Stokes radii. This was better visualized for both proteins when dephosphorylated by lambda phosphatase, which promoted smaller radii and, possibly, more compact or less hydrated conformations. It seems that an increase in phosphorylation induces a structural change that increases the apparent size or shape of hNek6. In fact, in most kinases, the activation loop is phosphorylated when the kinase is active, which stabilizes it in an open and extended conformation that is permissive for substrate binding [49]. This phosphorylated extended conformation may therefore contribute to the increase in hNek6 Stokes radius. All hNek6 variants were submitted to SEC-MALS twice, using two different buffers (the same one used for SAXS and another one containing 600 mM NaCl in order to avoid any unspecific binding to the gel filtration column resin), and the same Stokes radii for each protein were obtained in both measurements. Figure 6 shows the results from SEC-MALS using the buffer containing 600 mM NaCl, and Tables 2 and 3 show all the results obtained from both measurements.

**Figure 7:**
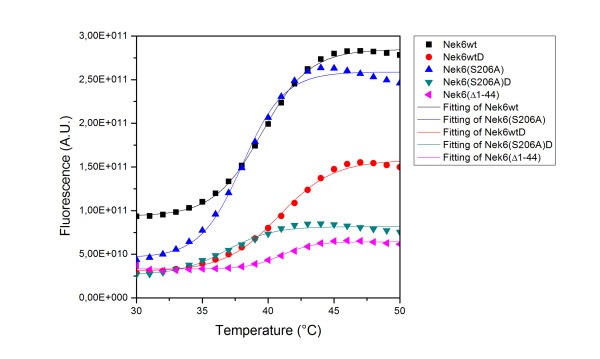
**Thermal shift denaturation of recombinant hNek6 variants fused to a 6xHis tag**. The graph shows representative thermal shift data (markers) and fitted curves (lines). Proteins were buffered in 10 mM HEPES pH 7.5, 150 mM NaCl and assayed at a final concentration of 2.0 μM in 25 μL volume. SYPRO-Orange (Molecular Probes) was added as a fluorescence probe at a dilution of 1 in 1000. Temperature was raised with a step of 1°C per 1.0 min from 10°C to 85°C and fluorescence readings were taken at each interval. (wt: wild-type, D: dephosphorylated).

Thermal denaturation shift assays were also performed for the five recombinant hNek6 variants described above. The results revealed a slightly higher stability of wild-type hNek6 compared to the activation loop mutant (Figure 7, Table 3). This may be explained by the fact that, in many kinases, like PKA, phosphorylation of the activation loop cause global stabilization of the active site [63], and molecular dynamics simulation of Cdk2 has demonstrated a decrease of B-factors throughout the molecule upon phosphorylation [64]. A model was proposed that dephosphorylation of the activation loop leads to mutual repulsion of the positive charges that were bound to the phosphate, which leads to the destabilization of the magnesium-binding loop, movement of the αC-helix out of the active site, disturbance of the hydrophobic spine, and loosening of the N-lobe, thereby providing an explanation of the protein kinase stabilization induced by phosphorylation [48]. This may therefore also reflect a higher stability for the wild-type hNek6 compared to its activation loop mutant in thermal melting measurements.

## Conclusions

Our data presents the first low resolution 3D structure of hNek6 protein in solution. SAXS experiments show that hNek6 is a monomer of a mostly globular, though slightly elongated shape, which was also confirmed by analytical SEC-MALS experiments. These also showed that hNek6 conformation is dependent on its activation/phosphorylation status, a higher phosphorylation degree corresponding to a bigger Stokes radius. Thermal denaturation shift assays revealed a slightly higher stability of wild-type hNek6 compared to the activation loop mutant hNek6(S206A).

## Methods

### *In silico *sequence analysis

Human Nek6 and Nek7 amino acid sequences were used as queries in five different secondary structure prediction databases: PredictProtein/Prof [29], PSIPRED [30], SSpro [31], SOPMA [32] and GOR4 [33]. Comparison of their outputs resulted in a consensus of predicted secondary structure, where each amino acid was assigned a score ranging from 1 to 5. Our Nek7 consensus of predicted secondary structure was compared to the author-approved secondary structure in PDB (2WQM) as a measure to validate our analysis. We also performed disordered regions analysis for both protein sequences using nine different predictors: FoldIndex [34], GlobPlot Russell/Linding [35], PONDR VL-XT [36], DISpro [37], IUPred [38], DisEMBL Hot-loops, DisEMBL Remark-465, DisEMBL Loops/coils [39], and VSL2B [40]. From this, a consensus of predicted disordered regions was generated with a consensus score ranging from 0 to 9, where a score above 4 represents disorder. Additionally, NetPhosK [42] and NetPhos [43] databases were used to predict phosphorylation sites for human Nek6 and Nek7. The conserved glycine-rich sequence, the HRD and DLG motifs, the conserved residues K^74 ^(β3 strand) and E^93 ^(αC helix), the putative nuclear export signal LGDLGL based on la Cour et al., 2004 [65], the putative WW domain binding motifs PY and pSP based on Ingham et al., 2005 [66], as long as the PPLP motif, experimentally described for hNek6 by Lee et al., 2007 [67], were also assigned to both protein sequences.

### Plasmid Constructions

All plasmid constructions were developed accordingly to Meirelles et al., 2010 [41].

### Site-directed Mutagenesis

The hNek6 activation loop mutation S206A was introduced by PCR-based mutagenesis accordingly to Meirelles et al., 2010 [41].

### Protein Expression and Purification

Soluble full-length hNek6 wild-type - 6xHis-hNek6wt - and mutant - 6xHis-hNek6(S206A) - or truncated hNek6 wild-type kinase domain - 6xHis-hNek6(Δ1-44) - fused to a 6xHis tag were expressed and purified accordingly to Meirelles et al., 2010 [41].

### Protein Dephosphorylation

In order to obtain dephosphorylated wild-type and mutant hNek6, plasmids encoding either 6xHis-hNek6wt or 6xHis-hNek6(S206A) and λ phosphatase were transformed into *E. coli *BL21 (DE3/pRARE) cells that were induced and purified as described by Meirelles et al., 2010 [41]. Lambda phosphatase cloned into pCDF-Duet (Novagen) was kindly provided by Dr. Richard Bayliss (Section of Structural Biology, Institute of Cancer Research, London, UK).

### Circular dichroism

Circular dichroism (CD) spectra were recorded in a JASCO model J-810 CD spectropolarimeter equipped with Peltier-type system PFD 425S. Data were collected from 260 to 200 nm at 4°C using a quartz cuvette of 1 mm pathlength. Thirty-two spectra of purified 6xHis-hNek6wt at 5.8 μM, in 50 mM Phosphate buffer pH 7.5, 300 mM NaCl, were averaged and corrected from the baseline for buffer solvent contribution. Experimental data were analyzed using CDNN [45] and K2d [46] softwares.

### Comparative/Homology Molecular Modeling

The comparative/homology molecular modeling and model validation were performed in a similar way to that described in Bodade et al., 2010 [68]. Briefly, several comparative/homology modeling tools were used: I-TASSER [69-71], Geno3D [72], 3D-JIGSAW [73-75], SWISS-MODEL [50] and MODELLER 9v8 [76]. The NCBI Basic Local Alignment Search Tool (BLAST, http://www.ncbi.nlm.nih.gov/BLAST/) was used to search the crystal structure of the closest homologue available in the Protein Data Bank (PDB, http://www.rcsb.org/pdb/). The input was the amino acid sequence of hNek6(S206A). The NCBI results revealed that the structure of hNek7, deposited under the code 2WQM in the PDB, was a very suitable template (identity score of 81% and E-value 3 × 10^-141^). This structure was used as a single template for the modeling approach. The overall stereochemical quality of the models was assessed by PROCHECK software [77]. The quality of the models was also evaluated by PROSA [51,52] and by the standard validation procedures included in the automated mode of the SWISS-MODEL server [50].

### Small Angle X-Ray Scattering Analysis

The sample was first inspected by dynamic light scattering (DLS) to test for its monodispersity and then ultracentrifuged at 200.000 × g for 40 min at 4°C to remove any possible aggregates. The SAXS experiments were performed at the D02A-SAXS2 beam line at the LNLS, and data treatment and analyses were done following standard procedures similar to those described in Trindade et al., 2009 [57]. Briefly, the measurements were performed at 4°C and the sample-to-detector distance was 902 mm, covering a scattering vector range of 0.015Å^-1 ^< q <0.25 Å^-1 ^(q is the magnitude of the **q**-vector defined by q = (4π/λ)sinθ and 2θ is the scattering angle) using a wavelength of λ = 1.488 Å. The measurements were performed using two different protein concentrations in HEPES buffer (50 mM HEPES pH 7.5, 5 mM sodium phosphate, 300 mM NaCl, 5% glycerol, 200 mM imidazole): 0.5 and 1.0 mg/mL. A 8 mg/ml BSA (66 kDa) solution in the same sample buffer was used as a standard sample to estimate the molecular mass of 6xHis-hNek6(S206A) making use of the ratio of the extrapolated values of the intensity at the origin, *I*(0) [78,79]. The radius of gyration (*R*_g_) was calculated from the Guinier approximation (valid for q*R*_g _< 1.3) [80-82] and also from the pair distance distribution function, *p*(r), which was obtained using the program GNOM [56]. The maximum dimension (*D*_max_) of the molecule was obtained from the *p(r) *function. The Kratky plot (*q*^2^I(*q*) vs. *q*) [81,82] was used to analyze the compactness of the protein conformation.

### Low resolution SAXS-based modeling

The low resolution model of 6xHis-hNek6(S206A) was obtained from the SAXS data using a combination of *ab initio *calculation and rigid body modeling methods. Taking advantage of the homology model obtained, we used the program BUNCH [61] to model the protein. No symmetry restraints were used in the calculation. We would like to mention that no unique solution can be obtained from these calculations. For this reason, 10 independent calculations were run for each sample data. The multiple solutions were analyzed and the reliability and stability of the set of models were estimated. A pairwise comparison and the normalized spatial discrepancy (NSD) evaluation was performed using the DAMAVER program suite [83] complemented by the SUPCOMB [84] routine. Analyzing the NSD values (which describe the dissimilarity between pairs of models of the several calculations), the models with common features led to the selection of a representative, low resolution conformation for hNek6(S206A) protein. Models were displayed by the PyMOL program [85].

For comparison purposes, two other low resolution models were also obtained by using two different *ab initio *approaches: the *dummy atoms *method implemented in the program DAMMIN [86] and the *dummy residues *method implemented in GASBOR [87]. The procedures were similar to those described in Trindade et al. [57].

### SEC-MALS Analysis

We used Analytical Size-Exclusion Chromatography coupled to Multi-Angle Light Scattering (SEC-MALS) to estimate the hydrodynamic or Stokes radii (*R*_s_) of recombinant hNek6wt, hNek6(S206A), hNek6(Δ1-44) and dephosphorylated hNek6wt and hNek6(S206A), all fused to a 6xHis tag. SEC was performed with an analytical Superdex 200 10/300 GL column using an ÄKTA FPLC system (GE Healthcare) equilibrated with two column volumes of 50 mM HEPES pH 7.5, 5 mM sodium phosphate, 600 mM NaCl, 5% glycerol, at a flow rate of 0.5 ml/min, at 20°C. Recombinant hNek6 variants at concentrations ranging from 0.2 to 0.7 mg/ml and a mixture of standard proteins with known Stokes radii (conalbumin: 3.64 nm, 3.2 mg/ml; ovalbumin: 3.05 nm, 4.2 mg/ml; carbonic anhydrase: 2.30 nm, 3.0 mg/ml; and ribonuclease: 1.64 nm, 3.4 mg/ml) (Sigma) were loaded onto the column and their elution profiles were monitored by absorbance at 280 nm. The Stokes radius of each hNek6 variant was estimated by a linear fit of the Stokes radii of the standard proteins versus the partition coefficient *K*_av _[88,89] as described by the equation: *K*_av _= *V*_e _- *V*_o_/*V*_t _- *V*_o _, where *V*_e _is the elution volume of the protein, *V*_o _the void volume and *V*_t _is the total volume of the column. The SEC was also coupled to a DAWN TREOS™ MALS instrument (Wyatt Technology). The on-line measurement of the intensity of the Rayleigh scattering as a function of the angle of the eluting peaks in SEC was used to determine the weight average molecular masses (*M*_w_) of the eluted proteins [90], using the ASTRA™ (Wyatt Technologies) software. SEC-MALS measurements were performed using two different buffers: the first one described above for SEC and a second one also used in SAXS experiments (50 mM HEPES pH 7.5, 5 mM sodium phosphate, 300 mM NaCl, 5% glycerol, 200 mM imidazole). The chromatographic profile of the recombinant hNek6 variants were the same in both measurements and the mean and standard errors of their *M*_w _and *M*_n _were calculated.

### Thermal Shift Assays

Thermal shift assays were performed based on a protocol devised by the Structural Genomics Consortium [91] using a real time PCR machine 7300 (Applied Biosystems). Proteins were buffered in 10 mM HEPES pH 7.5, 150 mM NaCl and assayed at a final concentration of 2.0 μM in 25 μL volume. SYPRO-Orange (Molecular Probes) was added as a fluorescence probe at a dilution of 1 in 1000. The emission filter for the SYPRO-Orange dye was set to 580 nm. Temperature was raised with a step of 1°C per 1.0 min from 10°C to 85°C and fluorescence readings were taken at each interval. OriginPro 8 software was used to fit data to the Boltzmann equation, y = LL+(UL-LL)/1+exp((*T*_m_-x)/a), where LL and UL are the slopes of the native and denatured baselines, *T*_m _is the apparent melting temperature and a describes the slope of the denaturation. *T*_m _values were calculated by determination of the maximum of the first derivative.

## Authors' contributions

GVM and JK conceived and designed the experiments, analyzed the data and wrote the manuscript. GVM performed the experiments. JCS performed comparative molecular modeling and SAXS experiments and interpreted them together with ICLT. YAM helped in SEC-MALS and CD experiments which were analyzed together with CHIR. JK supervised the project. All authors read and approved the final version of the manuscript.

## Supplementary Material

Additional file 1 **Supplemental Figures S1 and S2, PROCHECK and PROSA analysis results of the validation procedures of the hNek6(S206A) homology/comparative model and Analytical SEC-MALS of the five recombinant protein variants of Nek6**. Figure S1: PROCHECK and PROSA analysis results of the validation procedures of the hNek6(S206A) homology/comparative model. (A) Ramachandran Plot calculated using the program PROCHECK. (B) Plot of the residue score showing the local model quality by plotting energies as a function of the residue sequence position using PROSA. In general, positive values correspond to problematic or erroneous parts of the structure. Here, the plots were smoothed by calculation the average energy over 10- and 40-residues. This average is needed because of the large fluctuation in a plot of single residue energies. (C) The Z-score indicated overall model quality using PROSA. The Z-score of the hNek6(S206A) model was -7.14 (black point). The plot contains the Z-scores of all experimentally determined protein chains in the current PDB. The structure determined by X-ray and NMR are distinguished by different colors.  Figure S2: Analytical SEC-MALS of recombinant (A) hNek6wt, (B) hNek6wtD, (C) hNek6(S206A), (D) hNek6(S206A)D and (E) hNek6(Δ1-44). The Mw determined by MALS correspond to a monomer in all five cases. Click here for file
