# Supplementary material for: Human Nek6 is a monomeric mostly globular kinase with an unfolded short N-terminal domain
Source: BMC Struct Biol. 2011 Feb 14;11:12. doi: 10.1186/1472-6807-11-12 (PMC3053220; doi:10.1186/1472-6807-11-12)
Supplement: Additional file 1 — Supplemental Figures S1 and S2, PROCHECK and PROSA analysis results of the validation procedures of the hNek6(S206A) homology/comparative model and Analytical SEC-MALS of the five recombinant protein variants of Nek6. Figure S1: PROCHECK and PROSA analysis results of the validation procedures of the hNek6(S206A) homology/comparative model. (A) Ramachandran Plot calculated using the program PROCHECK. (B) Plot of the residue score showing the local model quality by plotting energies as a function of the residue sequence position using PROSA. In general, positive values correspond to problematic or erroneous parts of the structure. Here, the plots were smoothed by calculation the average energy over 10- and 40-residues. This average is needed because of the large fluctuation in a plot of single residue energies. (C) The Z-score indicated overall model quality using PROSA. The Z-score of the hNek6(S206A) model was -7.14 (black point). The plot contains the Z-scores of all experimentally determined protein chains in the current PDB. The structure determined by X-ray and NMR are distinguished by different colors. Figure S2: Analytical SEC-MALS of recombinant (A) hNek6wt, (B) hNek6wtD, (C) hNek6(S206A), (D) hNek6(S206A)D and (E) hNek6(Δ1-44). The Mw determined by MALS correspond to a monomer in all five cases. [file 1472-6807-11-12-S1.PDF]

Additional file 1

A

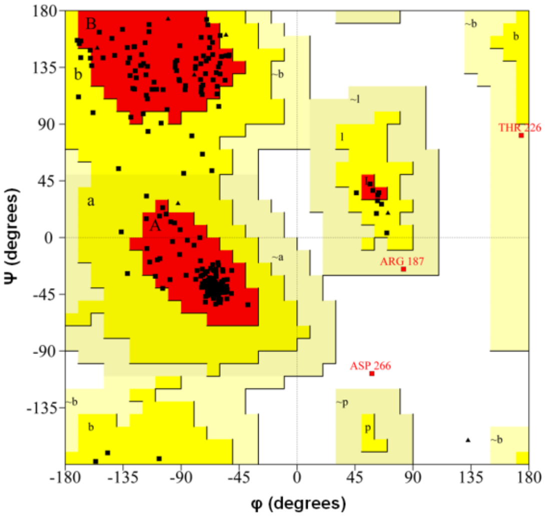

Ramachandran plot statistics

Residues in most favoured regions [A,B,L]	207	86.6%
Residues in additional allowed regions [a,b,l,p]	29	12.1%
Residues in generously allowed regions [~a,~b,~l,~p]	2	0.8%
Residues in disallowed regions	1	0.4%
	-------	---------
Number of non-glycine and non-proline residues	239	100%
Number of end-residues (excl. Gly and Pro)	2	
Number of glycine residues (shown as triangles)	14	
Number of proline residues	12	

Total number of residues	267	

B

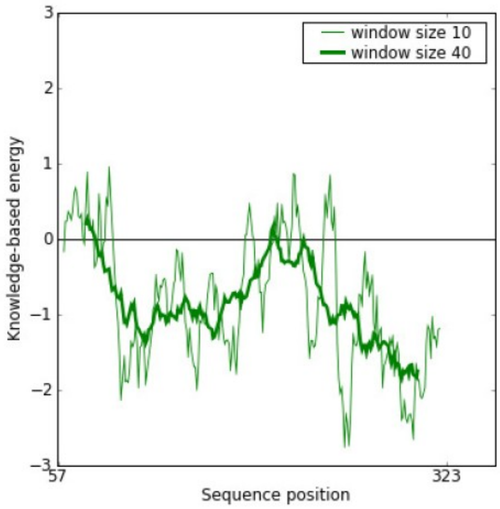

C

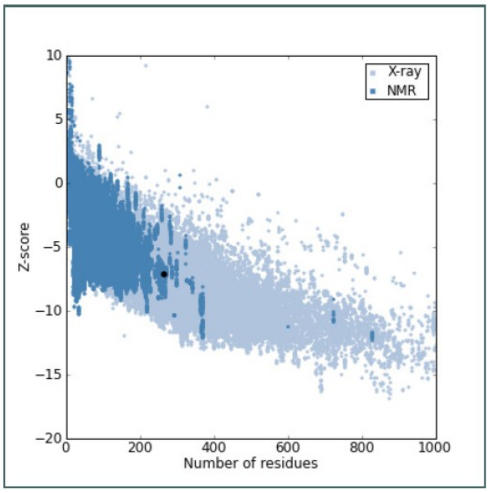

Figure S1. PROCHECK and PROSA analysis results of the validation procedures of the hNek6(S206A) comparative/homology model. (A) Ramachandran Plot calculated using the program PROCHECK. (B) Plot of the residue score showing the local model quality by plotting energies as a function of the residue sequence position using PROSA. In general, positive values correspond to problematic or erroneous parts of the structure. Here, the plots were smoothed by calculation of the average energy over 10- and 40-residues. This average is needed because of the large fluctuation in a plot of single residue energies. (C) The Z-score indicated overall model quality using PROSA. The Z-score of the hNek6(S206A) model was -7.14 (*black point*). The plot contains the Z-scores of all experimentally determined protein chains in the current PDB. The structures determined by X-ray and NMR are distinguished by different colors.

A

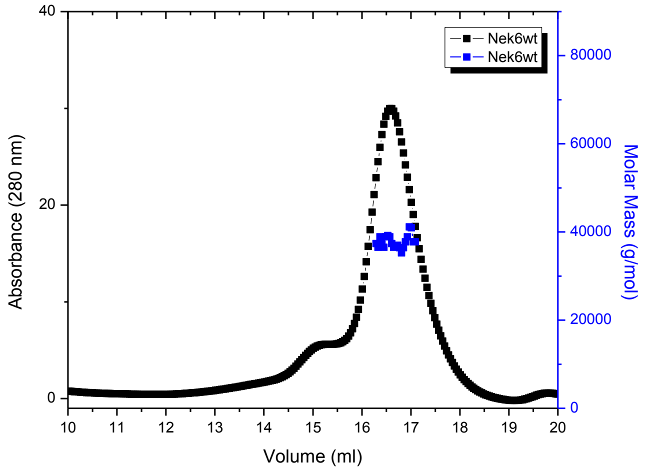

B

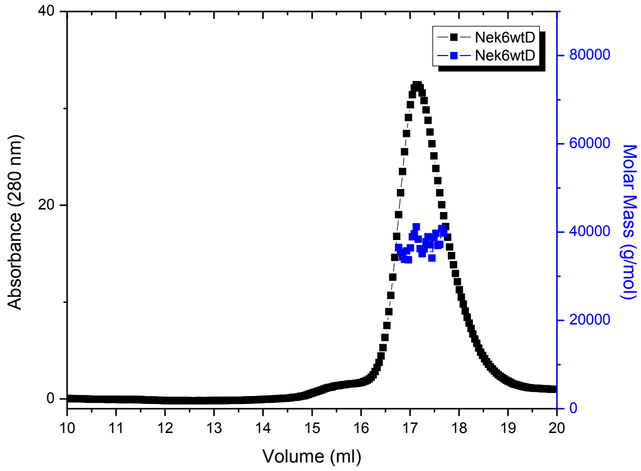

C

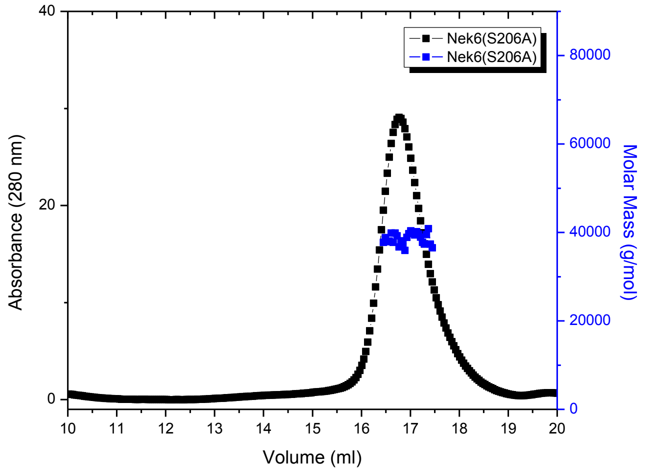

D

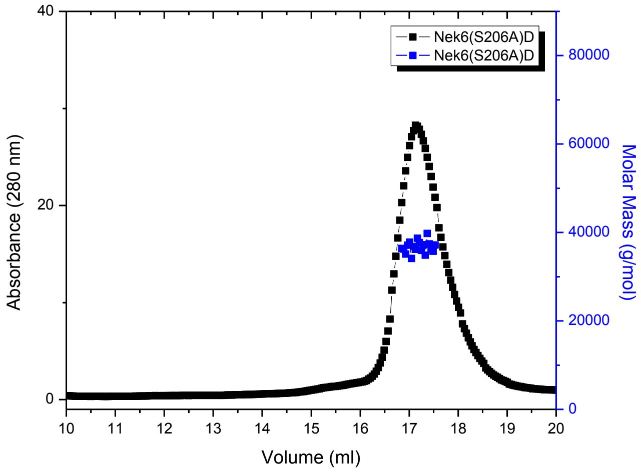

E

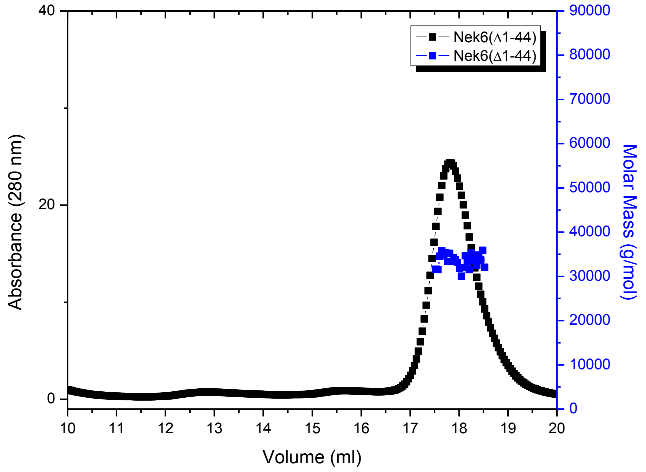

Figure S2. Analytical SEC-MALS of recombinant (A) hNek6wt, (B) hNek6wtD, (C) hNek6(S206A), (D) hNek6(S206A)D and (E) hNek6(Δ1-44). The M_w determined by MALS correspond to a monomer in all five cases.
